# Fabrication of Superhydrophobic Micro–Nanostructures on Pristine SLM-Ti Surfaces

**DOI:** 10.3390/mi17040454

**Published:** 2026-04-07

**Authors:** Xuetong Sun, Hao Sun, Xiue Ren, Changren Zhou

**Affiliations:** 1 College of Life Sciences, Zhuhai College of Science and Technology, Zhuhai 519040, China; 2 College of Chemistry, Jilin University, Changchun 130012, China

**Keywords:** superhydrophobic, selective laser melting, TiO_2_ nanotube, micro–nano structure

## Abstract

Superhydrophobic surfaces are typically achieved through the synergistic integration of appropriate nanostructures and low-surface-energy chemical compositions. This study presents a novel and facile method for constructing a superhydrophobic hierarchical structure directly on a pristine selective laser melting (SLM) titanium surface. The intrinsic partially melted Ti particles, which are inherent to the SLM fabrication process, were strategically utilized as a natural microscale template for the in situ growth of TiO_2_ nanotubes via electrochemical anodization. Three distinct micro/nano-topographies were successfully fabricated, integrating the spherical microparticles with either conventional TiO_2_ nanotube arrays or separated nanotube arrays. The results demonstrate that the resulting superhydrophobic behavior can be effectively regulated by two key factors: the liquid–solid contact mode at the microscale and the strength of capillary action within the nanostructures. Notably, these characteristics can be tailored by controlling the nanotube diameter and intertubular spacing. These findings contribute to a deeper understanding of the role of micro–nano hierarchical structures in engineering superhydrophobic surfaces, thereby opening new avenues for advanced applications.

## 1. Introduction

Inspired by natural architectures such as the self-cleaning lotus leaves and the directional water-repellency of butterfly wings, superhydrophobic surfaces have emerged as a pivotal area of materials science and surface engineering. These surfaces, typically defined by a water contact angle (WCA) greater than 150° and a sliding angle less than 10°, exhibit extreme water repellency. This unique property unlocks a vast array of transformative technological applications, ranging from self-cleaning windows and textiles [1,2] and anti-fogging optical lenses [3] to anti-biofouling coatings for marine hulls and antibacterial surfaces for medical implants [4,5], as well as spill-resistant protective gear and microfluidic devices. The fundamental origin of this exceptional wettability lies in the synergistic combination of two key factors: a hierarchical surface topography, featuring both micro- and nanoscale roughness, and a low-surface-energy chemical modification. This dual-scale architecture effectively traps air pockets beneath a water droplet, minimizing the liquid–solid contact area and resulting in the characteristic high contact angles and extreme droplet mobility, as described by the classic Cassie–Baxter model [6,7]. In recent years, various strategies have been developed to fabricate superhydrophobic surfaces with micro- and nanostructures that impart liquid-repellent functions [8,9,10].

Titanium (Ti) and its alloys stand as cornerstone materials in modern industry due to their exceptional combination of properties, including high specific strength, excellent corrosion resistance, and biocompatibility [11]. However, despite these advantages, titanium-based components face significant performance challenges that can drastically limit their service life and reliability. In biomedical settings, the native oxide layer on titanium, while biocompatible, is susceptible to bacterial adhesion and subsequent biofilm formation, leading to persistent infections and implant failure [12]. In harsh marine environments, titanium surfaces are prone to biofouling by marine organisms and accelerated corrosion in the presence of chloride ions and organic contaminants, compromising their structural integrity [13]. While fabricating superhydrophobic surfaces on titanium offers a promising route to mitigate these issues by reducing contact with corrosive media and preventing bacterial attachment, conventional modification approaches often fall short. Many existing techniques produce surfaces that, while hydrophobic, exhibit high water adhesion. This “sticky” superhydrophobicity (or parahydrophobicity) severely restricts practical utility in applications requiring self-cleaning or low drag, as the contaminants cannot be effectively carried away by moving droplets [14,15]. The challenge, therefore, lies in engineering a robust superhydrophobic state with low adhesion, which necessitates precise control over the surface’s hierarchical architecture.

This study aims to address this challenge by exploring a novel approach to construct a biomimetic hierarchical structure, directly leveraging the unique intrinsic features of additively manufactured titanium surfaces. Selective laser melting (SLM), a prominent laser powder bed fusion technique, has revolutionized the manufacturing of complex metallic components. The SLM process builds parts by selectively melting metal powder layers with a computer-controlled laser beam, enabling rapid fabrication of complex geometries [16,17]. A characteristic byproduct of this process, stemming from the partial melting of powder particles adjacent to the melt pool, is the inherent microscale roughness of as-built SLM surfaces. This roughness is predominantly dominated by the presence of numerous partially melted spherical Ti particles that are sintered or attached along the part contours [18,19]. While often considered a surface defect requiring post-processing, we recognize this inherent particle-decorated topography as a ready-made, naturally occurring microscale “template.”

Grounded in the established principles that surface roughness amplifies hydrophobicity and capillary effects modulate interfacial adhesion, we integrate micro-spherical Ti particles with either conventional TiO_2_ NTs or separated TiO_2_ SNTs to engineer SLM-Ti surfaces with tunable superhydrophobicity. The wetting behavior is primarily governed by the liquid–solid contact mode and the spatial configuration of entrapped air pockets. The micro/nano hierarchical structures are fabricated via electrochemical methods, with key morphological features—such as nanotube diameter, inter-tube spacing, and the morphology of the Ti microspheres—being controllable through adjustment of the electrochemical parameters. These structural variations alter the negative pressure associated with air pockets under different wetting regimes [20,21]. These findings provide new insights into the role of micro–nano hierarchical structures in regulating surface wettability and lay both a theoretical and experimental foundation for designing functional titanium-based surfaces tailored to diverse application requirements.

## 2. Materials and Methods

### 2.1. Materials

Square SLM-Ti samples (15 mm × 15 mm × 2 mm) were fabricated via SLM technique using a Concept Laser Mlab 200R system (Concept Laser, Lichtenfels, Germany), equipped with a 200 W Yb:YAG fiber laser featuring a spot diameter of 75 μm. Commercially available spherical pure titanium powder (>99.5% purity) with a particle size distribution of 20–40 μm was used as the feedstock. A 15 mm thick pure titanium plate served as the building substrate. The SLM process was conducted at a laser power of 200 W, a layer thickness of 25 μm, a scanning speed of 400 mm/s, and a hatch spacing (i.e., the distance between adjacent scan vectors) of 60 μm. The building chamber was evacuated and subsequently backfilled with argon prior to fabrication. Upon completion of the build, excess powder was removed from the sample surfaces. All samples were studied in their as-built state without any post-treatment, thus retaining their inherent surface roughness.

### 2.2. Fabrication of Micro–Nanostructures on SLM-Ti Surfaces

Micro–nanostructures were fabricated on SLM-Ti samples via electrochemical anodization using a platinum counter electrode under various conditions. Conventional TiO_2_ NTs were prepared by anodization at 15 V for 1 h in an electrolyte containing 0.3 wt% NH_4_F and 2 vol% deionized water. Separated TiO_2_ SNTs were synthesized by anodization at 40 V and 60 V for 40 min in an electrolyte composed of 0.5 wt% HF and 95 vol% diethylene glycol. The samples were subsequently modified with an ethanolic solution of 1H,1H,2H,2H-perfluorooctyltriethoxysilane (PTES, CF_3_(CF_2_)_5_CH_2_CH_2_Si(OCH_2_CH_3_)_3_, Aldrich). Specifically, 0.1 mL of PTES was dissolved in a mixture of 47.5 mL of ethanol and 2.5 mL of deionized water under magnetic stirring for 1 h. The SLM-Ti samples were immersed in the hydrolyzed PTES solution for 1 h to facilitate dehydration and condensation, then rinsed with ethanol, and finally heated at 100 °C for 2 h.

### 2.3. Characterization

The morphology of TiO_2_ NTs and SNTs on SLM-Ti substrates was examined using field emission scanning electron microscopy (FE-SEM, TESCAN MIRA, Brno, Czech Republic). Two-dimensional (2D) and three-dimensional (3D) optical profilometry was performed using a Leica DVM6 digital video microscope (Heerbrugg, Switzerland). Static, advancing, and receding water contact angles (WCA) were measured with an optical contact angle goniometer (SL200L2, KINO, Boston, MA, USA). For WCA and tilt angle measurements, water droplets of 5 µL and 7 µL were used, respectively. Each measurement was conducted on five independent specimens, with six distinct locations analyzed per specimen to ensure statistical reliability.

## 3. Results

**Figure 1:**
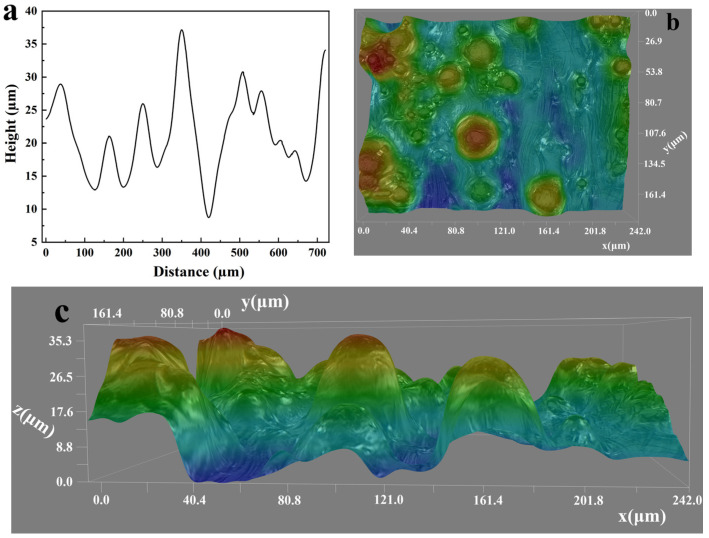
(**a**) 1D, (**b**) 2D, and (**c**) 3D surface profiles of the SLM-Ti substrate acquired using a digital microscope.

The 1D, 2D, and 3D surface profiles of the SLM-Ti substrate are presented in Figure 1. A substantial number of partially melted Ti particles were clearly observed to adhere to the substrate surface. The average peak height (Rp) and valley depth (Rv) were measured to be approximately 35.2 μm and 32.6 μm, respectively, based on five measurement points on each of the three independent specimens. Notably, this roughness scale is comparable to that of certain botanical leaf surfaces, such as lotus leaves [22].

**Figure 2:**
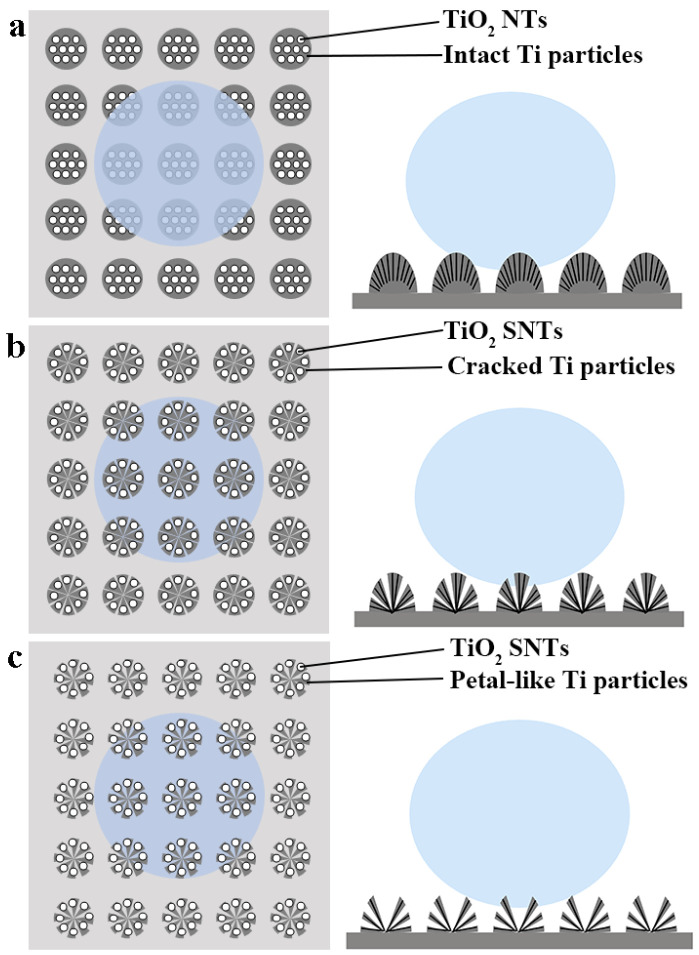
Schematics of three superhydrophobic micro–nanostructures. (**a**) Air trapped in conventional TiO_2_ NTs on intact SLM-Ti particles, featuring liquid–solid area contact. (**b**) Air trapped in separated TiO_2_ SNTs on cracked SLM-Ti particles, exhibiting liquid–solid line contact. (**c**) Air trapped in cracked, “petal-like” SLM-Ti particles, showing liquid–solid point contact.

Figure 2 illustrates the design strategies of three superhydrophobic micro–nanostructure models. A key characteristic of such surfaces is the creation of abundant and spatially confined air pockets—commonly termed Cassie–Baxter air traps—which constitute the fundamental physical basis for superhydrophobicity [23]. In general, the micrometer-scale architecture dictates the liquid–solid contact mode, thereby critically determining droplet adhesion strength. Importantly, a continuous three-phase (solid–liquid–gas) contact line (TCL) induces pronounced contact angle hysteresis (CAH) and strong pinning forces, whereas a fragmented TCL markedly suppresses both CAH and adhesion.

Guided by these principles, superhydrophobic micro–nanostructure models with tunable static and dynamic WCAs were developed. Figure 2a depicts micro-spherical Ti particles featuring numerous conventional NTs, wherein air pockets are encapsulated within the closed nanotubes, giving rise to an “area contact” mode. To minimize surface adhesion, the micro–nano structural design must preclude such enclosed air pockets, as they tend to intensify capillary-induced pinning. Optimal low-adhesion architectures instead target “line contact” and “point contact” modes, distinguished by minimal solid–liquid interfacial area and maximally fragmented TCLs [24]. Accordingly, micro-spherical Ti particles incorporating dispersed TiO_2_ NTs and exhibiting varying degrees of cracking were rationally designed (Figure 1b,c). The microscale petal-like morphology ensures highly localized, non-wetting contact points and extreme TCL discontinuity, thereby enabling spontaneous droplet roll-off with ultralow adhesion.

**Figure 3:**
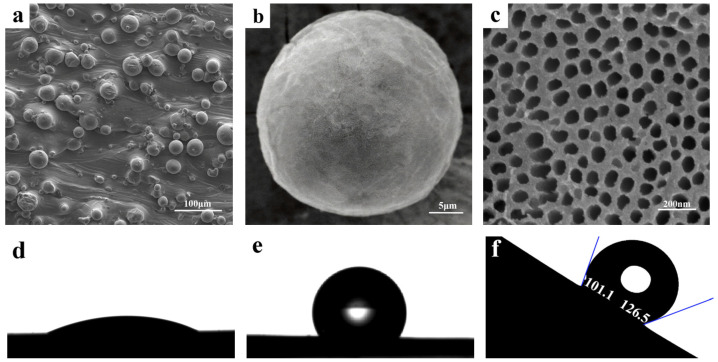
(**a**,**b**) SEM images of micro-sphere Ti particles with numerous TiO_2_ NTs structure (**c**), (**d**) a water droplet on the superhydrophilic TiO_2_ surface, (**e**) a droplet on the superhydrophobic PTES-modified TiO_2_ NPA structure and (**f**) a water droplet on a 45° inclined positioned superhydrophobic surface.

To demonstrate the effectiveness of the design strategies, three groups of micro–nanostructures were fabricated on the pristine micro-spherical particles on SLM-Ti surfaces using an electrochemical method. Figure 3a,b presents SEM images of a micro-spherical Ti particle composed of numerous TiO_2_ NTs (Figure 3d), obtained by anodizing an SLM-Ti sheet in 0.3 wt% NH_4_F electrolyte at 15 V for 1 h. A high density of well-ordered TiO_2_ NTs with diameters of 70.0 ± 3.5 nm are clearly visible on the surface. This nanotubular structure exhibited hydrophilicity (Figure 3d), attributed to its high surface energy and strong capillary adsorption effect [25,26].

To construct hydrophobic surfaces, the TiO_2_ NTs were modified with PTES. Figure 3e shows the shape of water droplets on the PTES-modified SLM-TiO_2_ NTs. After PTES modification, the water contact angle (WCA) of the SLM-TiO_2_ NTs increased from nearly 24.2° to 122.2°. The PTES-modified SLM-TiO_2_ NTs exhibit hydrophobic behavior, as the PTES molecules at the nanotube openings facilitate the formation of trapped (sealed) air pockets within the nanotubes [27]. As a result, the spherical droplet remained stably pinned on the surface with WCAs of θAdvancing/θReceding = 101.1°/126.5°, even when the substrate was tilted to 45° (Figure 3e).

**Figure 4:**
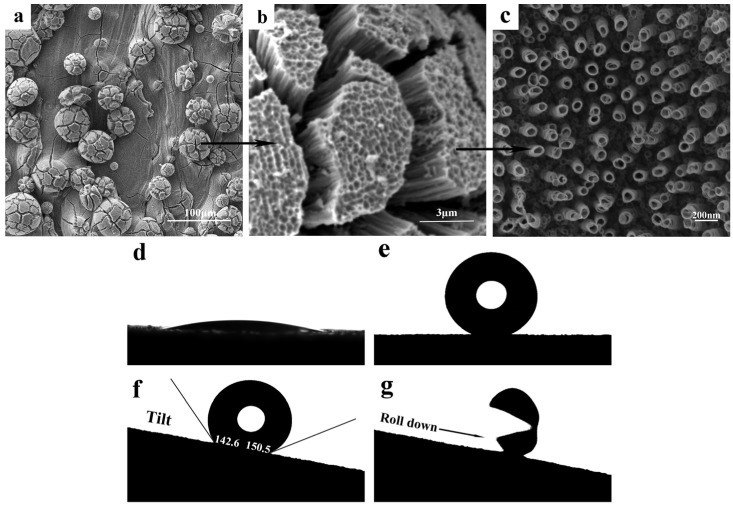
(**a**,**b**) SEM images of micro-sphere Ti particles with separated TiO_2_ SNTs structure (**c**), (**d**) a water droplet on the superhydrophilic TiO_2_ surface, (**e**) a droplet on the superhydrophobic PTES-modified TiO_2_ SNTs structure and (**f**,**g**) a water droplet on a 10° inclined positioned superhydrophobic surface.

Figure 4a–c shows SEM images of divided micro-spherical Ti particles composed of TiO_2_ separated nanotubes (SNTs), prepared in an electrolyte containing 0.5 wt% HF and 95 vol% diethylene glycol at 40 V for 1 h. The morphological transition is realized by using two distinct electrolyte systems. Conventional TiO_2_ NTs are synthesized via anodization in a low-viscosity ethylene glycol-based electrolyte at 15 V, wherein balanced oxide growth and field-assisted dissolution yield adherent, vertically aligned nanotube arrays. In contrast, TiO_2_ SNTs are fabricated in a high-viscosity diethylene glycol-based electrolyte under higher anodization voltages (40–60 V), where restricted ion transport promotes preferential lateral etching and induces interfacial stress accumulation at the nanotube–substrate interface, ultimately triggering spontaneous delamination of the nanotube layer.

The micro-spherical Ti particles consisted of TiO_2_ SNTs with an average diameter of 89 ± 5 nm and an intertubular spacing of 170 ± 5 nm. The spacing between these SNTs causes the complete micro-spherical Ti particles to split into petal-like architectures, with distinct gaps retained between adjacent petals. Following surface modification with PTES, the WCA of the SNTs nanostructure increased from nearly 8.6° to 151.8° (Figure 4d,e). Although both intact and cracked Ti particles are composed of TiO_2_ nanotubes, distinct structural differences exist: the microscale gaps in the cracked Ti particles promote the formation of additional trapped air pockets while eliminating capillary effects. In accordance with the Cassie–Baxter model, this results in an increase in WCA.

Under static conditions, both PTES-modified SLM-TiO_2_ NTs and SNTs surfaces exhibit hydrophobic characteristics. However, for applications requiring water repellency, droplet mobility (dynamic wetting behavior) becomes more critical [28]. Figure 3f shows the roll-off behavior of a water droplet on the tilted SLM-TiO_2_ SNTs surface, captured using a video contact angle instrument. As the SLM-TiO_2_ SNTs surface was gradually inclined from the horizontal plane to 10°, the water droplet slid off rapidly. Contact angle hysteresis (CAH), defined as the difference between the advancing and receding CAs, is a key parameter for evaluating superhydrophobic surfaces [29,30]. Typically, a surface is considered superhydrophobic when the static CA approaches or exceeds 150° and the CAH is lower than 5° [31]. In this study, the advancing and receding WCAs on the PTES-modified SLM-TiO_2_ SNTs surface were measured to be 150.5° and 142.6°, respectively, corresponding to a CAH of 7.9°—slightly above the conventional threshold for superhydrophobicity. As the air trapped between the nanotubes fails to form a sealed system capable of generating negative pressure, the total adhesive force is consequently reduced.

**Figure 5:**
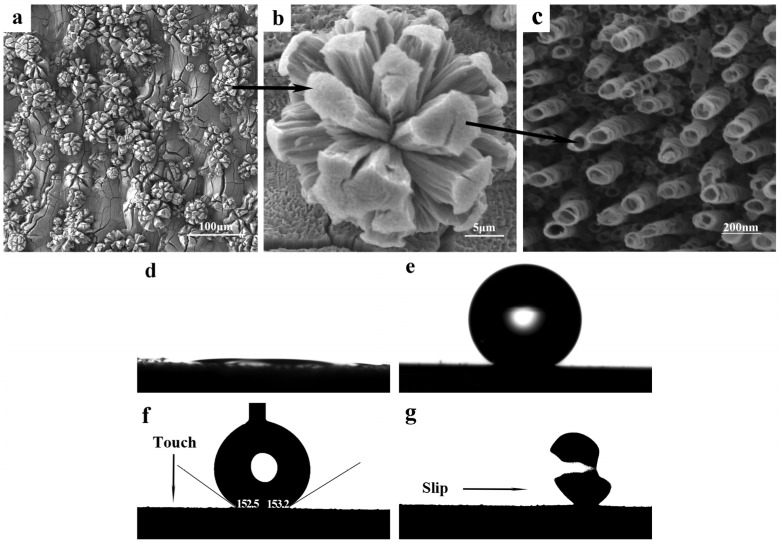
(**a**,**b**) SEM images of micro-sphere Ti particles with separated TiO_2_ SNTs structure (**c**), (**d**) a water droplet on the superhydrophilic TiO_2_ surface, (**e**) a roplet on the superhydrophobic PTES-modified TiO_2_ SNTs structure and (**f**,**g**) a water droplet on a horizontal positioned superhydrophobic surface.

Figure 5a,b presents SEM images of cracked “petal-like” Ti particles, which were fabricated at 60 V in a 95 vol% diethylene glycol electrolyte for 40 min. As shown in Figure 5c, these “petal-like” particles consist of TiO_2_ SNTs with an average diameter of 115 ± 5 nm and an average intertubular spacing of 260 ± 5 nm.

This unique “petal-like” microstructure satisfies the typical liquid–solid “point-contact” design requirements (Figure 2c), enabling the entrapment of extremely large air pockets and the formation of a highly discrete TCL [32]. Following modification with PTES molecules, the surface exhibited excellent superhydrophobicity with complete non-stickiness. The apparent WCA changed from 0° to approximately 155.0°, as illustrated in Figure 5e. Moreover, for the “petal-like” Ti particles composed of TiO_2_ SNTs, a water droplet moved spontaneously on the horizontal SLM-TiO_2_ SNTs surface (Figure 5f,g), with almost no CAH, exhibiting advancing and receding angles of 152.5° and 153.2°, respectively.

**Figure 6:**
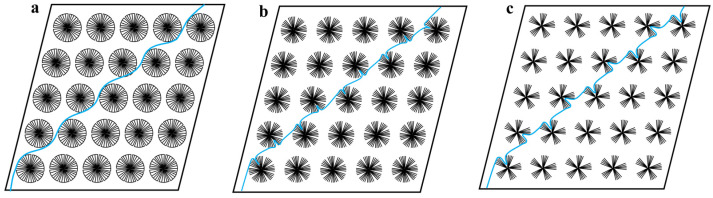
Schematic illustration of the surface topography: (**a**) conventional TiO_2_ NTs on intact SLM-Ti particles, (**b**) separated TiO_2_ SNTs on cracked SLM-Ti particles, (**c**) separated TiO_2_ SNTs on “petal-like” SLM-Ti particles. Blue lines denote the theoretical three-phase contact lines of a sessile water droplet on each respective surface.

Figure 6 illustrates the three-phase contact line of a water droplet on TiO_2_ NTs and TiO_2_ SNTs surfaces. The blue lines represent a possible three-phase contact line, positioned to maximize the contact area with the surface. For the TiO_2_ NTs sample, the droplet diameter is on the millimeter scale, while the TiO_2_ nanotubes are on the nanometer scale; therefore, the contact line at the roughness scale appears nearly straight when viewed at the droplet diameter scale. The contact line may become pinned due to continuous and stable solid–liquid contact (see Figure 6a). Accordingly, the TiO_2_ NTs surface exhibits advancing and receding water contact angles of *θ_Advancing_*/*θ_Receding_* = 101.1°/126.5°. In contrast, for the TiO_2_ SNTs surfaces, the three-phase contact line in Figure 6b,c is more distorted and less continuous, resulting in reduced solid–liquid contact and increased contact with air [33,34]. In this configuration, the droplet does not remain pinned prior to advancing or receding, but instead moves spontaneously, exhibiting almost no contact angle hysteresis.

## 4. Conclusions

This study presents a straightforward and viable approach for fabricating biomimetic superhydrophobic microstructures on SLM-Ti surfaces. Experimental results demonstrate that superhydrophobic behavior can be effectively modulated by the solid/air/liquid contact mode and the spatial configuration of trapped air pockets, both of which are governed by the morphology of microscale Ti particles. These particle configurations can be further tailored by adjusting the diameter of TiO_2_ nanotubes and the intertubular spacing. Notably, on “petal-like” particle surfaces assembled from TiO_2_ SNTs, water droplets were observed to move spontaneously across a horizontal surface with nearly zero CAH. These findings offer new insights for the rational design of advanced superhydrophobic surfaces, enabling fine control over the microstructural features of metal particles on additively manufactured metal substrates.

## Data Availability

The raw data supporting the conclusions of this article will be made available by the authors on request.
